# What makes partnerships work? Measuring agreement in participant beliefs regarding ‘core’ reasons for success

**DOI:** 10.1371/journal.pone.0356371

**Published:** 2026-09-18

**Authors:** Mason Clay Mathews, Alexandria J. Drake

**Affiliations:** 1 Spatial Data Science Center (SDSC), Department of Geography, Florida State University, Tallahassee, Florida, United States of America; 2 Master of Public Health Program, University of Puget Sound, Tacoma, Washington, United States of America; Access Alliance Multicultural Health and Community Services: Access Alliance, CANADA

## Abstract

Successful partnerships between people with different backgrounds and distinct beliefs are essential to solve 21st-century problems. We conducted 44 semi-structured interviews and 80 surveys with participants of academic-nonprofit partnerships. We used Plackett-Luce statistics and cultural consensus analysis to analyze respondent beliefs regarding partnership success. Respondents consistently placed a high value on a subset of ‘core’ reasons that academic-nonprofit partnerships succeed and exhibited higher-than-expected levels of agreement regarding the order of importance of these ‘core’ reasons. The approach we present here can be used before, during, and after partnerships to improve the design, implementation, and evaluation of team science.

## Introduction

As hubs of innovation and knowledge creation, academic institutions are increasingly expected to collaborate with partners in industry, government, nonprofit, and community sectors to solve complex problems. Millions of dollars and thousands of hours of research time have been invested in cross-sectoral and cross-disciplinary research programs focused on innovation and knowledge production [1,2]. These resources can be squandered if the partnerships upon which these efforts are built are poorly conceived, designed, and implemented.

A recurring finding in academic partnership research is that different work and organizational cultures can affect partnership outcomes [3–6]. For example, there is evidence that ‘ivory tower’ academics and ‘real world’ people in nonprofits, industry, schools, and government struggle to collaborate due to conflicting values, goals, and methods, which can “unnecessarily inhibit the forging of strong work relationships” [7]. Here, we measure the degree to which people participating in academic-nonprofit partnerships share beliefs regarding what causes these partnerships to succeed.

In this analysis, we address the following research questions:

(i) What value or ‘worth’ do academic-nonprofit partnership participants place on the different steps, processes, and activities that lead to partnership success?
(ii) Do the participants in academic-nonprofit partnership share beliefs about the order of importance of the steps, processes, and activities that lead to partnership success?
(iii) Do subgroups of academic-nonprofit partnership participants hold distinct shared beliefs regarding the order of importance of the steps, processes, and activities that lead to partnership success?

### Literature review

There is an extensive body of scholarly research regarding academic partnerships with communities, industry, nonprofits, and government organizations [8–10]. Scholars have produced numerous studies (and review articles) regarding how and why these partnerships succeed and fail [9,11,12]. Several studies focus on how organizational cultures can shape partnership outcomes [13–15]. Building on this work, we set out to ***measure*** the degree to which participants ***share beliefs*** regarding why academic-nonprofit partnerships succeed.

#### The importance of partnerships in 21st-century research.

Partnerships that bring together the specialized skills, knowledge, and resources of people from academia, industry, nonprofits, governments, and communities are essential to solving the complex ‘wicked’ problems of the 21st century [16,17]. These problems include, but are not limited to, climate change, biodiversity loss, homelessness, and income inequality.

Since the 1990s, academic institutions have faced increasing demands to go beyond traditional research and teaching to engage in ‘third mission’ activities that address complex societal problems [18,19]. ‘Third mission’ activities and research require non-academic knowledge and skills and depend upon partnerships that involve local communities, industry, nonprofits, and governments [20].

Convergence research is a 21st-century paradigm that involves partnerships between people and organizations with distinct backgrounds that span disciplinary boundaries [21]. Combining their unique skills and expertise enables convergence researchers to create innovative solutions for ‘wicked’ problems, such as climate change mitigation [22], community resilience [23], and hazards and disaster management [24], that require experts from different disciplinary areas.

Efforts to understand the dynamics of increasingly diverse research groups have coalesced into a research effort known as the Science of Team Science (SciTS). SciTS studies have shown that interdisciplinary teams are more likely to be highly productive and generate novel, high-impact research than lone scholars and teams with homogeneous backgrounds [25–27].

‘Third mission’, convergence, and the multi, inter, and transdisciplinary research studied by SciTS scholars depend on effective partnerships involving diverse actors with distinct backgrounds, motivations, and belief systems. The degree to which participants acknowledge, respect, and understand one another’s culture and belief systems can profoundly shape partnership outcomes.

#### Culture in partnerships.

When people share beliefs, values, and norms, they are said to have a common ‘culture’ [28]. Shared cultural models play a pivotal role in people’s understanding of the world and their behavior in it [29]. The role of culture in shaping partnership outcomes is a common thread across the different disciplines that study partnerships [15,30–32].

Although team diversity is considered essential to solving complex problems, it does not come without its own set of challenges. Experts with diverse backgrounds and expertise may have very different beliefs regarding how partnerships should work. Cultural differences can cause clashes regarding partnership goals, objectives, time schedules, activity calendars, and desired products and outcomes [33].

People involved in academic-nonprofit partnerships may come from different organizational cultures [34], with diverse workplace incentives and behavioral norms, speak different technical languages, and may even believe stereotypes about, or harbor resentment towards, one another [30,35]. Subcultures within groups may also have different belief systems and motivations. For example, students, staff, and professors belonging to the same academic culture may have distinct subgroup beliefs regarding partnerships [36].

It is often assumed that people understand partnerships in similar ways, such as opportunities to ‘work together’ [37]. However, people typically bring both their individual *and* organizational cultural models to partnerships [30]. These combinations of cultural models can lead to diverse beliefs regarding the importance of the different interactions, steps, and processes necessary for effective collaboration. If participants hold different beliefs regarding these processes, emphasizing some while deemphasizing others may lead to conflicts that negatively affect the partnership.

#### Distributed and team cognition in partnerships.

Cultural beliefs are just one type of shared cognition that can shape partnership outcomes. Team cognition theory argues that cognition occurs at the team level, where members collectively develop and share understandings, knowledge, and expertise, which they use to achieve collective goals and objectives [38,39]. In teams and partnerships, team cognition shapes members’ capacity to acquire, distribute, store, and retrieve critical knowledge [40]. Distributed cognition theory argues that cognitive processes, such as thinking, memory, and beliefs, are not restricted to individuals but are system-level processes shared across groups of people, environments, tools, and time [41].

Team and distributed cognition are manifested through shared mental models (SMMs) [42]. Team performance scholars use SMM theory to explain team dynamics and outcomes [43]. Gevers et al. [44] found that when team members shared beliefs about their collaborative tasks and timelines, it boosted their collective confidence and enhanced team performance. Measuring and understanding SMMs in teams and partnerships can help decipher how members share crucial information, where in the team unique knowledge resides, and how members act as a unit rather than as individuals [45].

#### Partial and overlapping shared mental models.

In some circumstances, people who do not share complete mental models may share beliefs about subcomponents of these models. For example, using cultural consensus and residual agreement analysis, Dengah [46] found that while people from two Pentecostal sects in Brazil largely agreed on some ‘core’ elements of what constituted a ‘complete life’, their beliefs diverged on the importance of others.

People engaging in partnerships may share subsets of beliefs that shape their behaviors in important ways, even when they do not share complete mental models. In our example here, ‘core beliefs’ refer to a subset of elements that a cross-section of people consistently deemed important for academic-nonprofit partnerships to succeed. Identifying these ‘core’ beliefs can serve as a starting point to define partnership goals and to design, plan, and implement the interactions, steps, and processes necessary for partnership success. Here we present an approach that can be used to identify shared beliefs *and* subsets of shared ‘core’ beliefs regarding elements that may be essential for partnership success.

### Data collection

Our research effort included six phases of mixed methods data collection. This section explains each phase in detail, including our decision-making processes and how these steps and decisions shaped our final results.

Phase 1 – Literature review. Beginning in 2020, we conducted an extensive literature review of peer-reviewed articles, books, and grey literature, including how-to guides, focused on academic-nonprofit, academic-industry, academic-government, and academic-K12 partnerships. Our literature review indicated that although some partnership beliefs span the different types of academic partnerships, some beliefs may not. We focused our primary data collection on academic-nonprofit partnerships to avoid a potential mix of belief systems associated with distinct partnership types.

Phase 2 – Cultural domain analysis (Semi-structured interviews). In Phase 2, we conducted semi-structured interviews to conduct a cultural domain analysis of an emic/insider understanding of how academic-nonprofit partnerships work. Following Weller [47], we designed the cultural domain analysis to inform the development of our cultural consensus analysis survey in phase four. For example, we asked informants to freelist reasons for partnership success and then explain their importance for partnerships as suggested by Borgatti [48].

Following Handwerker’s [49] sampling guidelines for cultural domain analysis research, we used a purposive, snowball sampling approach to recruit people with academic-nonprofit partnership experience. We employed Maximum Variation Sampling [50] to capture a wide range of partnership beliefs. For example, we interviewed students, staff, and professors, as well as nonprofit CEOs, directors, and staff. Between August 2021 and June 2022, we conducted forty-four semi-structured interviews via Zoom, which we recorded and transcribed. Prior to being interviewed, all participants received the project consent form via email, which we reviewed again with the participants before each interview began. All participants provided verbal consent before recording and questions commenced. This consent process followed the protocol approved by the Office of Research Integrity and Assurance at Arizona State University. This study was determined to be exempt pursuant to Federal Regulations 45CFR46 [2].

Phase 3 – Qualitative coding and thematic analysis. We coded the semi-structured interview transcripts in MAXQDA. First, we consolidated the respondents’ freelists and answers for each question. Second, we grouped the similar list items and answers into themes. Finally, we created a comprehensive list of all the potential question responses from our semi-structured interviews and literature review. Our coding analysis enabled us to turn qualitative partnership expert’s cultural domain data into a list of reasons academic-nonprofit partnerships succeed. S1 Table A includes our final list of the reasons academic-nonprofit partnerships succeed and the number of semi-structured interview participants who mentioned each reason. This table also includes examples of publications from our literature review that contain mentions of the reasons for partnership success that people freelisted in the semi-structured interviews.

Phase 4 – Survey development and cognitive interviewing. In Phase 4, we developed an online survey instrument to measure shared beliefs regarding the themes and topics we identified in Phases 1–3. Our literature review and semi-structured interviews indicated people’s partnership beliefs might be heavily influenced by their organizational culture (academic vs nonprofit), their organizational status (student vs non-student), and their experience working on academic nonprofit partnerships. For this last trait, we asked three partnership experience questions, 1) the number of academic nonprofit partnerships in which respondents had participated, 2) the number of years they had worked on these partnerships, and 3) the types of organizations they worked in when engaged in these partnerships. During our semi-structured interviews, we discovered that people had often worked on partnerships in academic organizations *and* nonprofits at different stages in their careers. Therefore, we added a question asking if respondents had experience working on partnerships in academic organizations, nonprofits, or both. Table 1 describes the respondent attribute data from our survey.

**Table 1:** Respondent Attributes.

Attribute	Variables	n
1. Gender	Female	61
	Male	18
	Non-binary	1
2. Student Status	Non-student	60
	Student	20
3. Organization Type	Academic Organization	45
	Nonprofit Organization	27
	Other	8
4. Experience Type	Academic Experience	21
	Nonprofit Experience	25
	Both Experience	34
5.Number of Partnerships	Above Median Number of Partnerships (>=4)	46
	Below Median Number of Partnerships (<4)	31
	Missing Data Median Number of Partnerships	3
6. Years Partnerships	Above Median Number of Years Partnerships (>=5)	41
	Below Median Number of Years Partnerships (<5)	39
	Total Respondents	80

Once the preliminary survey was completed, we conducted cognitive interviews via Zoom with a six-person panel over three sessions between January and April 2023. Following Willis [51], we created a protocol of cognitive interview questions to determine how the panelists understood each survey question and the descriptions of the rank-order items. Cognitive interviewees received IRB-approved consent form emails and provided verbal consent prior to the interviews. After the third round of cognitive interviews, we concluded that we had improved the survey to the best of our ability.

Phase 5 – Survey data collection. Our final survey contained seven rank-order questions regarding partnership beliefs and the aforementioned demographic and partnership experience questions. The latter were collected as potential explanatory variables that might be associated with shared beliefs regarding partnerships and are summarized in Table 1. We conducted our survey via the Qualtrics online survey platform. The list of rank order items was randomized in Qualtrics for each survey to avoid instances of false agreement. Respondents used the interactive features in Qualtrics to drag items into the order of their choosing.

Since cultural consensus analysis is used to measure cultural patterns of shared beliefs, respondents must have some experience with the cultural domain being modeled [47,52]. Therefore, scholars use non-random, purposive samples for CCA survey data collection [49,53]. Using a purposive sampling approach, we identified survey respondents who had participated in at least one academic-nonprofit partnership. We used Maximum Variation Sampling [50] to obtain a survey sample representative of the different subgroups that participate in academic-nonprofit partnerships, including students, staff, professors, and nonprofit CEOs. Our recruitment emails were approved by the Office of Research Integrity and Assurance at Arizona State University, as was the research explanation and consent statement at the beginning of the survey. Between May and October of 2023, we collected 80 online surveys via Qualtrics.

Phase 6 – Survey data analysis. The final phase included cleaning the completed survey data, creating a codebook, and conducting statistical analyses. All data in this study were deidentified to protect the participants’ anonymity. The methods section below explains our choice of statistical methods and how they work. In the results section, we present our research findings for the rank-order question in our survey focusing on the reasons partnerships succeed.

## Methods

We used several statistical measures, including Plackett-Luce statistics, in conjunction with cultural consensus analysis (CCA) to analyze respondents’ answers to our ranking question about the causes of academic-nonprofit partnership success. Our goal with this multi-step analysis analytical triangulation was to determine if any patterns of partnership beliefs appeared across different statistical tests.

### Plackett-luce statistics: Measuring preferences

The Plackett-Luce Model (PLM) combines the work of Luce [54] and Plackett [55]. Luce’s choice axiom states that the relative odds of choosing one item over another from a group of items are not affected by the presence or absence of the other group items [54]. Plackett [55] defined probability distributions for rank-order data, introduced parameters to characterize the probability distributions, and developed a parameter estimation method.

Based on Luce’s axiom, the rank ordering of the items is seen as a sequence of choices from the items remaining [56]. When an individual ranks a set of items from most to least important, they select the most important item from those that have yet to be ranked [57]. The Plackett-Luce statistic measures each item’s ‘worth’ parameter in a set of rank-order items based on how respondents rank them [58]. Items that respondents rank higher will also have a higher ‘worth’ value [59]. The Plackett-Luce model establishes a reference category to obtain the statistical significance of differences between item strength/worth [59]. This reference category can be one of the items or the mean item ‘worth’ [58].

### Cultural consensus analysis (CCA): Measuring shared beliefs

While Plackett-Luce statistics are useful to identify item-by-item patterns in rank-order data, cultural consensus analysis (CCA) is used to determine if people **share beliefs** regarding answers to a set of questions [52,60]. Cultural consensus methods can be used to understand widely held cultural beliefs, such as what constitutes a ‘good life’ in Brazil [61], as well as shared beliefs among subgroups, such as how different Brazilian Pentecostal denominations understand what it means to be a ‘true believer’ [46]. Management and organization studies scholars have used CCA to measure shared knowledge and cultures in organizations [62], to identify ‘operational subcultures’ [63], and to compare international beliefs regarding cooperation [64].

Cultural consensus analysis measures shared beliefs by first creating an agreement matrix of the correlations of responses to survey questions [52]. Each matrix cell captures the correlation between two respondents’ answers. Next, eigenfactor decomposition (similar to factor analysis) is run on the agreement matrix to determine if there is a strong agreement in the form of a single-factor solution [52]. Next, a competence score is calculated for each respondent by comparing their answers with those of the group [47]. This score, typically between 0 and 1, captures the percentage of each respondent’s answers that agreed with the group’s ‘culturally correct’ answers [65]. The model also produces a 2^nd^ factor loading score for each respondent, capturing variation beyond the first factor [47]. These scores can be used to understand residual agreement, which may exist between members of respondent subgroups [66]. Finally, the model produces an answer key by weighting each respondent’s answers by their competency score [52]. The answer key is deemed to represent the most agreed-upon answers to the CCA questions [67]. By convention, three diagnostics indicate if shared beliefs exist among respondents: 1) the eigenvalue ratio between the first and second eigenvalues is greater than 3:1, 2) there are few, if any, negative competence scores, and 3) there is a mean competence score of 0.5 or greater [47].

Cultural consensus models are calculated differently depending on the type of questions used to collect data. The informal model accepts ranked, interval, or ratio-scaled response data [47]. In this model, an agreement matrix is factored, and the competence scores are used to weight respondent’s responses (by multiplication), which are then summed together [47].

**Fig 1:**
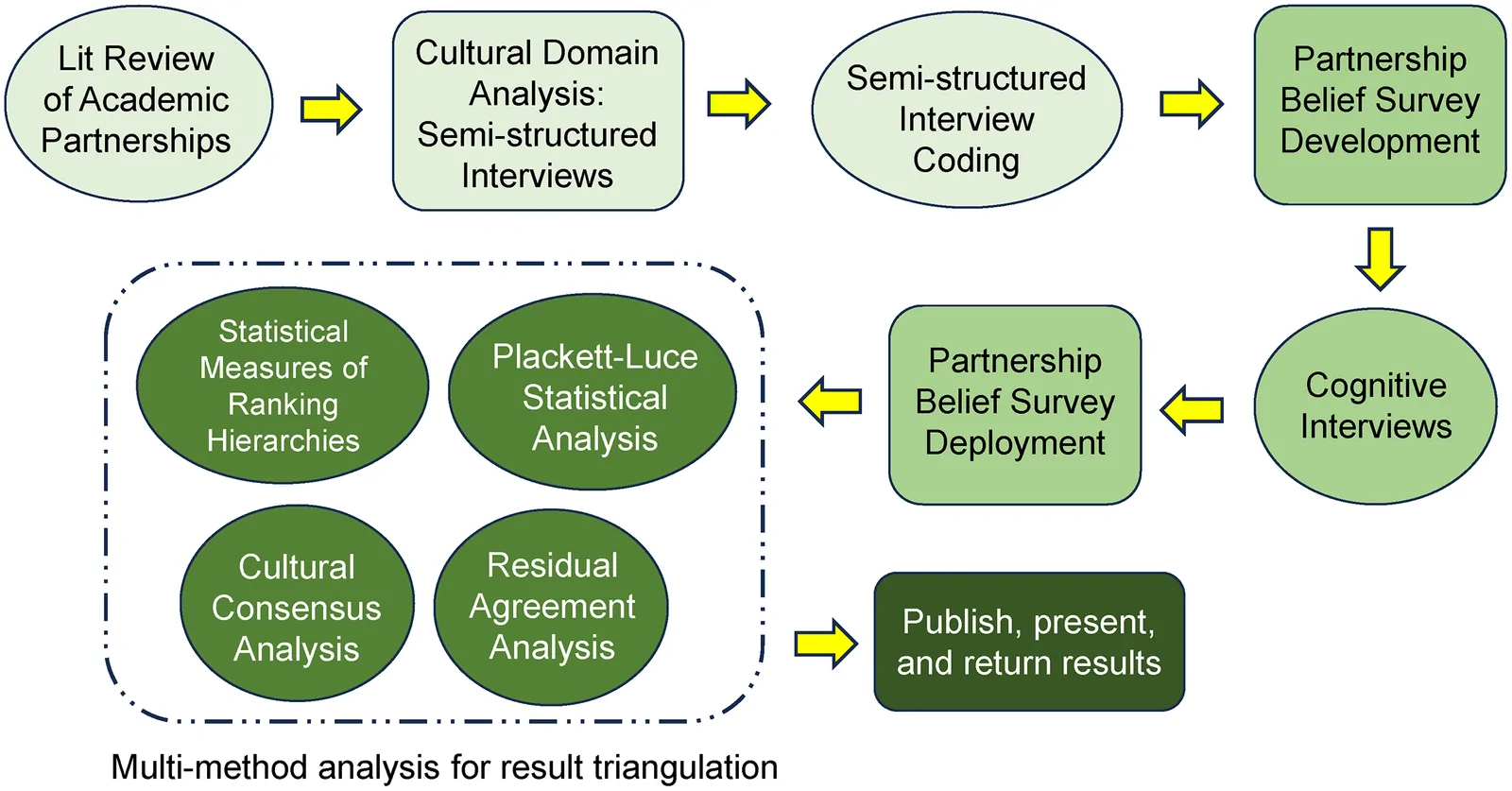
Research roadmap to measure shared and ‘core’ partnership beliefs. This figure provides a visual of project workflow.

We decided that multiple-choice, Yes/No, or True/False questions might not be very illuminating. For example, measuring whether people agree that effective communication is important for partnership success might not advance our understanding of partnerships. Instead, we used a rank-order question format to determine whether respondents shared beliefs regarding the importance of reasons, like effective communication, compared to other reasons for success. Fig 1 shows our research workflow, which includes data analysis methods that when used together, make it possible to identify shared beliefs *and* subsets of ‘core’ beliefs. We have not seen this set of statistical methods used together to determine the ‘worth’ people place on reasons for partnership success and the degree to which groups share these beliefs.

## Results

In this section, we present the results from our analysis of question 10 in our survey, which asked respondents to rank order the reasons academic-nonprofit partnerships succeed. In this question, respondents were asked to rank order the 18 items in S1 Table A from most to least important as reasons for academic-nonprofit success. The reasons are also listed in Table 2.

a. Statistical analyses of ranking hierarchies

We used Kendall’s coefficient of concordance to measure the consistency of rankings across the 80 survey respondents, Kendall’s W(80, N = 18) =  .254, χ2 (17) = 345.7, p < 2.2e-16. The results indicated a level of ‘fair agreement’ according to the interpretation levels suggested by Landis and Koch [68]. However, it is important to note that Kendall’s W tests absolute, group-wide concordance in exact ranking and may not capture agreement regarding subgroups of ranking items.

We conducted a Friedman rank sum test to determine if respondents showed a significant preference among the 18 items. The results indicated a statistically significant difference in the rankings across the items, χ^2^ (17) = 345.7, p < 2.2e-16. Since the Friedman test is global, we also ran a post hoc pairwise comparisons Conover test with a Bonferroni correction. The p-value table from this test (S1 Table B) shows which ranking pairs had means that were statistically different (<0.05) and which did not (>0.05).

**Fig 2:**
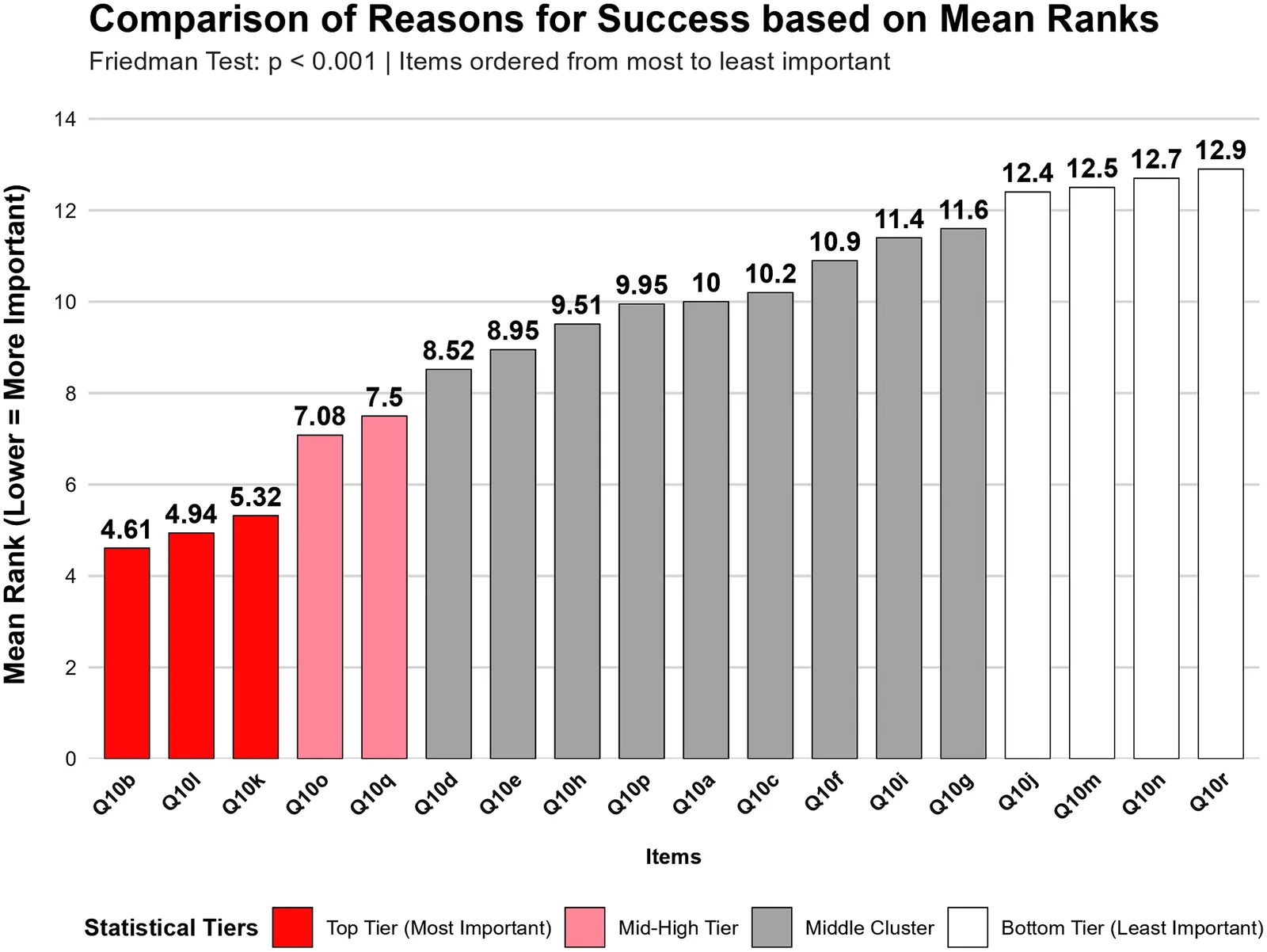
Friedman test and mean ranks comparison. This figure highlights mean comparisons for reasons for partnership success based on survey results.

Pairing the pairwise comparison p-value table (S1 Table B) with the mean rankings (Fig 2), revealed a definitive hierarchy in the importance respondents placed on different reasons for academic-nonprofit partnership success. Q10b, Q10k, and Q10l were highly unique; they had extremely small p-values <0.05 (S1 Table B), compared to the other reasons for success, indicating that respondents ranked them differently. These reasons also had the lowest mean rankings (Top Tier in Fig 2), indicating they were consistently ranked as more important for partnership success. A marginal ranking table (S1 Table C) corroborates this result, Q10b was ranked in the top three by 46 out of the 80 respondents (58%) and Q10l was ranked number one by 29 out of the 80 respondents (36%).

Together, the post hoc p-values and mean ranks suggest Q10o and Q10q sit in a Mid-high Tier (Fig 2) of reasons that were considered less important than the top three, but more important than the rest of the reasons. The Middle Cluster (Fig 2) includes reasons that were largely statistical ties when compared to one another in the pairwise analysis, indicating a lack of agreement regarding which were more important. The marginal ranking table (S1 Table C) corroborates this finding, Q10h for example, received similar votes for all the ranking choices. Finally, there was a Bottom Tier (Fig 2) of reasons that were statistically indistinguishable from each other in the pairwise comparisons and had the highest mean ranks, meaning they were consistently ranked lower than the other reasons (S1 Table B).

The statistical tests in this section indicate that respondents shared mental models regarding the ‘core’ importance of communication, vision, and goals reasons for success (Q10b, Q10l, and Q10k) while agreeing slightly less regarding the importance of establishing clear roles and obtaining sufficient funding (Q10o and Q10q).

b. Plackett-Luce item ‘worth’

The summary in Table 2 includes the ‘worth’ estimates, standard errors, and associated hypothesis test statistics and p-values for each item when the mean ‘worth’ is used as the reference. Here, a higher item ‘worth’ indicates that a reason was more likely to be ranked higher as something that leads to partnership success. The top five reasons (Q10b – Q10q) had statistically significant positive estimates that indicate that they were all ranked higher than the mean ranking (9.5). The following five reasons (Q10e – Q10c) in Table 2 had statistically non-significant positive and negative estimates that were neither above nor below the mean ranking. The bottom eight reasons (Q10p – Q10r) had statistically significant negative estimates, indicating they were consistently ranked lower than the mean ranking. Lower ranked items should not be interpreted as unimportant for partnership success. Our rank order question required respondents to prioritize reasons for success, revealing patterns in which items were generally given a higher or lower priority as reasons for success rather than distinguishing between important and unimportant factors.

**Table 2:** Plackett-Luce Statistical Results – ‘Worth’ / Importance of Reasons Partnerships Succeed.

ID	Q10 Reasons academic-nonprofit partnerships succeed	Estimate	Std. Error	z value	Pr(>|z|)	Prob Rank 1	Mean Rank
Q10b	Clear partnership and project goals, expectations, deliverables, and outcomes	1.16197	0.12417	9.358	<2e-16 ***	0.14562	4.61
Q10k	Regular and clear communication	1.09655	0.11854	9.25	<2e-16 ***	0.1364	5.32
Q10l	Participants have a shared partnership vision/purpose	1.03776	0.12291	8.443	<2e-16 ***	0.12861	4.94
Q10o	Clear partner roles and responsibilities	0.54798	0.11902	4.604	4.14e-06 ***	0.07881	7.08
Q10q	Sufficient funding to complete partnership activities and tasks	0.29338	0.11998	2.445	0.014476 *	0.0611	7.50
Q10e	Shared decision-making opportunities	0.13404	0.1178	1.138	0.25518	0.0521	8.95
Q10d	Administrative buy-in and support from decision-makers	0.1098	0.11966	0.918	0.358804	0.05085	8.52
Q10a	Realistic timelines to complete partnership tasks	−0.01072	0.11528	−0.093	0.925898	0.04508	10.00
Q10h	Shared feelings of influence and power in partnership decisions	−0.06191	0.11925	−0.519	0.603674	0.04283	9.51
Q10c	Equitable distribution of partnership benefits	−0.19012	0.11869	−1.602	0.109177	0.03767	10.24
Q10p	Partnership champion (e.g., an influential person who can advocate for the partnership with decision-makers)	−0.32032	0.12279	−2.609	0.009091 **	0.03307	9.95
Q10f	Having a system to monitor and measure partnership activities	−0.31243	0.11769	−2.655	0.007936 **	0.03334	10.91
Q10i	Equitable investment of resources	−0.39258	0.11785	−3.331	0.000865 ***	0.03077	11.45
Q10g	Equal distribution of tasks and responsibilities among partners	−0.40061	0.11745	−3.411	0.000648 ***	0.03052	11.59
Q10n	Partnership risks are shared equitably	−0.54419	0.11703	−4.65	3.32e-06 ***	0.02644	12.68
Q10j	Clear partnership duration and end date	−0.6955	0.12663	−5.492	3.97e-08 ***	0.02273	12.38
Q10m	Co-developed partnership language that avoids jargon	−0.72825	0.12527	−5.813	6.12e-09 ***	0.02199	12.50
Q10r	Establishing a co-created conflict resolution plan	−0.72485	0.12134	−5.974	2.32e-09 ***	0.02207	12.88

Signif. codes: 0 ‘***’ 0.001 ‘**’ 0.01 ‘*’ 0.05 ‘.’ 0.1 ‘ ’ 1.

Residual deviance: 5485.2 on 12223 degrees of freedom.

AIC: 5519.2.

Number of iterations: 6.

These Plackett-Luce estimates and Z-values suggest that respondents believed that the top four items in Table 2 (Q10b, Q10k, and Q10l) were the most important reasons for partnership success. These reasons centered on clear goals and objectives, effective communication, and shared vision/purpose. Rounding out the top five were clear roles/responsibilities (Q10o) and sufficient funding (Q10q). This Plackett-Luce item ‘worth’ analysis illuminates respondents’ value systems regarding the individual reasons for partnership success. While the differences between the top five reasons were modest, the differences between the top and bottom reasons in Table 2 were significant. The Plackett-Luce estimates, z-values, and probability of ranking first measures (Table 2) corroborate the findings of the Friedman rank test, marginal ranking table, and post hoc pairwise comparisons, revealing a clear preference for the Top Tier and Mid-High Tier reasons for success in Fig 2. They refine the analysis of the Middle Cluster by comparing the ranked items to the mean center (9.5), which separates items in the neutral center from those trending downwards. This explains the difference between the Middle Cluster in Fig 2 and Table 2. The Plackett-Luce results also reiterate the levels of agreement regarding reasons that were consistently ranked less important for partnership success in the other tests (Bottom Tier Fig 2).

We can evaluate the performance of the Plackett-Luce model (PLM) using the residual deviance statistic [58], which for this model was 5485.2 with 12,223 degrees of freedom. The deviance-to-df ratio was 0.45. A deviance-to-df ratio well below 1.0 implies underdispersion in the model, meaning that there are *fewer* differences between the observed data and fitted probabilities than expected [69]. This may be due to the strong agreement across the sample of the importance of the Top Tier reasons for partnership success that were especially evident in the marginal ranking table, mean ranks, and post hoc pairwise comparisons. However, we also recognize that our sample size and sampling approach may have implications for our statistical results (see limitations section below). Nevertheless, given that the PLM estimates largely agree with the mean rankings and pairwise comparisons, the model appears to capture the basic hierarchical trends in the respondents’ partnership success beliefs.

### Cultural consensus analysis (CCA) and shared partnership beliefs

a. Shared partnership beliefs and residual agreement among subgroups

We used cultural consensus analysis to test whether the results of respondents’ rankings exhibited patterns of shared beliefs regarding the order of importance of reasons academic-nonprofit partnerships succeed. First, it is important to note the likelihood of respondents ranking the reasons for success in the same order. The possible rankings for these items are 18 factorial (18!), which equals a staggering 6.402374e + 15 or 6.4 quintillion possible ranking combinations. With 80 respondents and such a large number of possible ranking orders (1/18!)^80, the likelihood of 80 respondents ranking all 18 items the same by chance is astronomically small. With so many possible ranking options and such low probabilities of ranking items in the same order by chance, we expected little if any agreement in the rankings.

The ratio between the first and second eigenvalue for the cultural consensus model with all 80 respondents was 2.96, with a mean competence score of  .49, and zero negative competencies. We used UCINet 6 Version 6.801 to run the consensus models [70]. Although the model meets the suggested minimum threshold of 3:1 for eigenvalue ratios and >.50 for competence scores [47,71], it is important to note that this model hovers right at the minimum threshold. Eigenvalue ratios below 3:1 may indicate that there is no single, shared cultural model among the respondents, or that subgroups within the sample hold distinct or conflicting beliefs [72]. Given this possibility, we tested whether subgroups in the sample exhibited higher agreement levels.

T-tests with the binary demographic variables from Table 1 and the respondents’ competence scores indicated that some subgroups had different levels of agreement with the overall sample of respondents (see Table 3). The t-test for gender did not reveal a significant difference in the mean competence scores for men and women. We removed the single non-binary respondent from this analysis. The ANOVAs for Organization Type and Partnership Experience Type were not statistically significant, indicating that the levels of agreement of respondents in these subgroups regarding reasons for partnership success did not vary widely from the overall sample.

**Table 3:** ANOVA and t-test results of competence scores.

Attribute	Results
1. Gender (n = 79)	Welch Two Sample t-test t = -0.8245 df = 27.702 p-value = 0.4167 95 percent confidence interval: -0.1736470 0.0740107 sample estimates: mean in group Male mean in group Female 0.4558164 0.5056346
2. Student Status (n = 80)	Welch Two Sample t-test t = 3.1158 df = 27.697 p-value = 0.004241 95 percent confidence interval: 0.06409906 0.31046490 sample estimates: mean in group Non-Student mean in group Student 0.5397049 0.3524230 Cohen’s d estimate: 0.8964815 (large) 95 percent confidence interval: lower upper 0.3634337 1.4295293
3. Organization Type (n = 80)	ANOVA Df Sum Sq Mean Sq F value Pr(>F) Q2OrgType 2 0.087 0.04343 0.87 0.423 Residuals 77 3.843 0.04991
4. Experience Type (n = 80)	ANOVA Df Sum Sq Mean Sq F value Pr(>F) Q3ExpType 2 0.119 0.05973 1.207 0.305 Residuals 77 3.811 0.04949
5. Number Partnerships (n = 77)	Welch Two Sample t-test t = -2.5489 df = 60.57 p-value = 0.01336 95 percent confidence interval: -0.23234333 -0.02803968 sample estimates: mean in group Below Median mean in group Above Median 0.4195981 0.5497896 Cohen’s d estimate: -0.6026944 (medium) 95 percent confidence interval: lower upper -1.0756074 -0.1297813
6. Years Partnerships (n = 80)	Welch Two Sample t-test t = -2.3243 df = 76.038 p-value = 0.02278 95 percent confidence interval: -0.21011440 -0.01619613 sample estimates: mean in group Below Median mean in group Above Median 0.4348924 0.5480476 Cohen’s d estimate: -0.5213559 (medium) 95 percent confidence interval: lower upper -0.97415911 -0.06855262

However, the t-tests for the student status and partnership experience variables identified statistically significant differences in mean competence scores for these groups (Table 3). Non-students had higher mean competence scores, indicating that their rankings were more strongly aligned with the shared beliefs of the entire respondent group. The Cohen’s d effect for this test was large, suggesting that students and non-students understood the reasons partnerships succeed differently. This result may have several causes and implications. First, the nature of our sampling approach (purposive snowball), led to a sample of students of different age groups, partnership experience, and disciplinary focus areas. This diversity may have led to the lack of agreement in the student subgroup. Answering questions regarding differences in beliefs between students and non-students requires replicating this research in different settings (see next steps below).

T-tests for the number and years of partnership experience (Table 3) indicated that respondents with more partnership experience had higher average competence scores, which suggests that their rankings were more strongly aligned with those of the entire respondent group. However, the Cohen’s d effects for these t-tests were medium, the p-values were not strongly significant, and the difference in the means was not large.

b. Residual agreement results

Since the competence scores indicated the possibility of residual agreement beyond the first factor, we ran several residual agreement tests. First, following Boster [73], we calculated a predicted agreement matrix for the entire sample and subtracted it from the observed agreement matrix to generate a residual agreement matrix. We then ran a QAP correlation of the observed and residual matrices. The correlation was  .752 with a p-value of 0.000. When the observed and residual agreement matrices are correlated, the overall group consensus does not exhaust the residual agreement, and subgroup belief variations may be present [66].

Next, following Boster and Johnson [74], we plotted the competence scores and the 2^nd^ factor loading scores (S1 Fig–S6 Fig). We generated plots for each respondent attribute collected in the survey that may explain subgroup variation. To interpret the plots, we turn to the residual agreement framework created by Henderson et al. [72]. Given that the respondent competence scores (S1 Fig–S6 Fig) cluster along the 1^st^ factor (x-axis) and are dispersed along the 2^nd^ factor (y-axis), we appear to have a multicentric cultural domain with overall consensus [72]. This means that while there is overall group consensus (as indicated by the 2.96 eigenvalue for the overall data), the agreement pattern tends to be stronger within residual agreement groups [72].

To better understand the subgroup consensus, we first ran cultural consensus models for each of the subgroups. Table 4 contains the cultural consensus results for subgroups in our data that exhibited some shared beliefs. First, we can see that the eigenvalue ratios all crossed the 3:1 threshold, with mean competence scores > .50 threshold. The competence score standard deviations were not small, indicating that agreement was unevenly distributed, even within the context of overall agreement. The range of the competence scores was wide, indicating variation between the beliefs of some respondents and the overall group. Finally, there was only one negative competence score, indicating that only one respondent disagreed with one model.

**Table 4:** Cultural Consensus and Academic-Nonprofit Partnership Respondent Subgroups.

Subgroup	1^st^ to 2^nd^ Eigenvalue ratio	Mean cultural competence (± SD)	Range of cultural competence/Neg. competencies
Non-student (n = 60)	3.115	.54 (± .20)	.05 to .89 / 0
Non-profit Non-student (n = 26)	3.004	.55 (± .18)	.06 to .89 / 0
Experience Type Both (n = 34)	3.197	.54 (± .20)	.10 to .90 / 0
Experience Type Both Non-student (n = 28)	3.601	.55 (± .21)	.17 to .92 / 0
Above Median Number Partnerships (n = 46)	3.344	.55 (± .21)	.01 to .89 / 0
Above Median Number Yrs Partnerships (n = 41)	3.344	.54 (± .22)	−.02 to .88 / 1

To better measure agreement across subgroups, we compared the answer keys from all the subgroup CCA models to determine if (and how) they were similar. First, we created a heat map of the subgroup answer keys for side-by-side comparison (Table 5), which reveals several consistent patterns. First, the heat map color intensity shows similar high rankings across answer keys for the ‘core’ reasons from the Top Tier and Mid-High Tier reasons in (Fig 2. And Table 3), which were consistently ranked the most important. Second, we also see similar rankings across answer keys for the lowest-ranked reasons. Third, the heat map shows that the most divergent answer key belonged to the students, who disagreed with the other subgroups regarding the importance of administrative buy-in (Q10d) and partnership champions (Q10p). Although the answer keys varied across some rankings, which explains the different competence scores among subgroups, the ‘core’ reasons for success remained remarkably consistent across subgroup answer keys.

**Table 5:** Cultural Consensus Subgroup Answer Key Rankings Heat Map.

Item	A*	NS*	S	AO	NO	ANS	NNS*	AEO	NEO	BE*	BENSTO*	BENSAO	AMN*	BMN	AMNY*	BMNY
Q10a	10.5	10.6	9.1	10.5	10.4	10.8	10.4	10.2	9.8	10.9	11.1	11.3	10.9	9.6	10.7	10.1
**Q10b**	3.5	3.4	4.4	3.6	3.1	3.7	3.1	3.3	3.4	3.6	3.3	3.5	3.5	3.3	3.2	3.8
Q10c	10.7	10.7	11.8	11.2	10.3	11.3	10.3	10.8	12.0	9.9	9.8	11.7	10.5	11.3	11.4	9.9
Q10d	7.4	6.8	11.1	7.9	7.6	7.0	7.6	8.2	7.5	7.1	6.8	8.1	7.0	8.8	6.8	8.4
Q10e	9.0	8.8	9.0	8.8	8.7	8.6	8.7	7.9	8.6	9.6	9.0	8.9	8.8	9.4	9.1	8.9
Q10f	11.4	11.3	11.3	11.6	10.5	11.6	10.5	11.5	9.6	12.4	12.5	11.9	11.6	11.3	10.6	12.4
Q10g	12.4	12.4	11.3	12.5	12.2	12.4	12.2	13.5	12.2	11.7	11.7	10.9	12.4	11.8	12.6	11.9
Q10h	9.7	9.8	9.6	9.2	10.2	9.2	10.2	8.1	10.0	10.3	10.3	10.4	9.9	9.7	10.1	9.4
Q10i	11.7	11.8	12.3	11.7	12.5	12.1	12.5	13.7	12.1	10.5	10.8	10.5	11.7	11.9	12.0	11.2
Q10j	12.6	12.9	11.3	12.8	11.8	13.6	11.7	11.9	11.3	13.8	13.6	12.9	13.2	11.3	13.1	12.1
**Q10k**	5.5	5.6	4.5	5.3	5.4	5.6	5.4	5.4	5.2	5.6	6.0	5.8	5.4	5.7	5.6	5.1
**Q10l**	3.7	3.5	4.7	3.9	3.1	3.7	3.1	4.4	3.9	3.3	3.3	3.4	3.1	4.1	3.5	3.6
Q10m	12.7	12.8	12.0	12.1	14.1	12.0	14.1	11.0	13.7	12.7	12.4	12.2	12.7	13.2	12.3	13.3
Q10n	12.9	13.1	12.0	12.4	12.8	12.6	12.9	12.9	13.0	12.9	13.4	12.5	13.2	12.0	13.7	11.8
**Q10o**	6.6	6.5	6.4	6.9	6.1	6.7	6.1	6.3	6.9	6.6	6.2	6.6	6.1	7.3	6.4	6.8
Q10p	10.1	9.4	13.1	10.6	10.1	9.2	10.1	11.3	9.7	9.9	9.2	8.9	9.6	11.2	8.7	12.0
**Q10q**	7.0	7.3	6.5	6.5	8.3	7.0	8.4	7.7	8.2	6.0	6.7	6.5	7.4	6.5	7.0	7.4
Q10r	13.8	14.2	10.8	13.2	13.8	13.8	13.8	12.7	13.7	14.1	14.8	14.9	14.1	12.7	14.4	12.7

Subgroup Answer Keys: A* = All NEO = Nonprofit Experience Only. NS* = Non-student. BEO* = Both Experience Red Item ID **=** Top Tier reason for partnership success. S = Student BENSTO* = Both Experience Non-student Only Purple Item ID = Mid-High Tier reason for partnership success. AO = Academic Organization. BENSAO = Both Experience Non-student Only. NO = Nonprofit Organization. AMN* = Above Median Number of Partnerships. ANS = Academic Non-student. BMN = Below Median Number of Partnerships. NNS* = Nonprofit Non-student. AMNY* = Above Median Number of Years Partnerships. AEO = Academic Experience Only BMNY = Below Median Number of Years Partnership. (A * in the column header indicates an eigenvalue > 3:1 for that subgroup).

Notably, the answer key analysis results mirrored the semi-structured interview data; the top three reasons for success were the most frequently free-listed (S1 Table A) and consistently ranked the highest. We also ran a Kendall’s Tau correlation of the answer keys, which revealed a great deal of agreement across the answer keys, except for the student answer key (S1 Table D).

Finally, following Dengah and Dressler [65,66], we created divergence plots to determine the degree to which the rankings of the reasons for success varied across subgroups (S7 Fig–S14 Fig). In this approach, positive subgroup rankings have higher deviation scores (e.g., 1,2,3) in the plot, and lower-ranked items have lower deviation scores (e.g., −1, −2, −3) [46]. Items with no deviation between the subgroup and overall respondent sample rankings lie at the plot origin (0,0) [46]. We created plots for each of the different subgroups in Table 4. In these plots, we colored the Top Tier ‘core’ reasons for success in red and the Mid-High Tier in pink. Although there is some variation across subgroups (particularly the student vs non-student plot), the Top Tier ‘core’ reasons hovered around the origin in each plot, which reconfirms the finding that the Top Tier (communication, vision, and goals) reasons were consistently ranked highly as important for partnership success. The Mid-High Tier reasons fluctuated much more, indicating less consistency in respondent’s shared mental models regarding clear roles (Q10o) and sufficient funding (Q10q) than those regarding the Top Tier reasons for partnership success.

## Research limitations

A mix of statistical analyses are useful to verify the existence of patterns, such as the consistent importance of ‘core’ beliefs, in data. However, challenges can arise when statistical approaches have different assumptions regarding sampling and other data characteristics. Given that our cultural consensus survey questions required a non-random, purposive sample, we do not make any claims regarding the inferential power of our statistical measures to explain wider beliefs regarding partnerships beyond our sample. This will require replication, which we address in the discussion section. In addition, while 80 respondents may be more than adequate for many cultural consensus analysis studies, a larger sample may be necessary for a rank order question with 18 items to produce robust statistical findings for the other analyses used here.

We conducted this research while working at a university which emphasizes partnerships with non-academic organizations, prizes community engagement, and has offices that help connect academics with community partners. Since these conditions may have shaped our research outcomes, we believe it is necessary to replicate this research in other settings to determine if our findings are more widely generalizable (see discussion).

### Results summary

In this study, we used semi-structured interviews with academic-nonprofit partnership participants to generate a list of reasons partnerships succeed from domain experts. A combination of analyses including Friedman’s test, mean ranks, post hoc pairwise comparisons, and a marginal ranking table identified a set of ‘core’ reasons partnerships succeed. Plackett-Luce statistics captured the ‘worth’ that participants assigned to each reason for partnership success. Finally, we used cultural consensus analysis to measure partnership participants’ shared beliefs regarding the order of importance of the reasons academic-nonprofit partnerships succeed.

The statistical tests and CCA analysis answer keys all indicated that respondents placed a high value on communication, vision, goals, roles, and funding as reasons academic-nonprofit partnerships succeed. The residual agreement divergence plots indicate that these ‘core’ reasons for success were less likely to diverge in subgroup beliefs (S7 Fig–S14 Fig). These ‘core’ reasons for success appear to be foundational processes upon which partnerships can be built. For instance, once everyone agrees on what a partnership is about (Q10l), what everyone will do (Q10o), and when they will do it (Q10b), other steps can be taken. Clear communication (Q10k) is essential to organize the ‘what,’ ‘who,’ and ‘when,’ and to make adjustments as the partnership unfolds. Sufficient funding (Q10q) is also an important ingredient for success, although respondents’ beliefs regarding funding were not as consistently shared as their beliefs about the other ‘core’ items.

In response to the research questions at the beginning of this article, we can say the following. First, respondents in our sample consistently placed a high value or ***‘worth’*** on a ‘core’ set of reasons that academic-nonprofit partnerships succeed (Fig 2, Table 2). Second, respondent rankings exhibited higher-than-expected amounts of shared belief regarding the order of importance of a subset of ‘core’ reasons partnerships succeed. Third, although academic and nonprofit respondent subgroups from distinct organizational cultures ranked some reasons differently, they agreed with the overall respondent group regarding the importance of the ‘core’ reasons for academic-nonprofit partnership success.

## Discussion

The innovation necessary to solve complex 21^st^-century challenges requires partnerships that bring together people with diverse skills, knowledge, and expertise. These people often come from distinct organizational cultures and may understand partnerships differently. The degree to which participants share beliefs and mental models regarding which steps, processes, and activities lead to success may shape partnership outcomes.

Our exploratory research indicates that people may share a subset of ‘core’ beliefs regarding how partnerships work, even when they do not share complete mental models. If this finding is repeated with additional research, it raises new questions that merit further exploration. For example, do beliefs regarding similar ‘core’ reasons for success exist across different partnership types? If so, is it enough for people to share ‘core’ beliefs in order for partnerships to succeed? How do we leverage this knowledge to design better partnerships?

Our research shows how mixed data analysis methods can be combined to measure shared mental models in partnerships. However, there are numerous possible next steps. This research could also be replicated with different types of academic partnerships (industry, K12, community, government) to determine if similar belief patterns emerge. In fact, this approach could be replicated across different types of partnerships (e.g., business, diplomatic, family, etc.) to determine if people share beliefs regarding a set of ‘core’ reasons that ***all*** partnerships succeed.

Upon reflection, an additional step of reviewing the statistical results with research participants could enhance this research. In this follow-up, researchers could ask participants why they ranked certain reasons for partnership success higher (or lower), whether they agree (or not) that certain steps are more important for partnership success, and why this is the case. These follow-up interviews can serve as an opportunity to return research results to participants and as a way to gain information that can help interpret the statistical results. For example, follow-up questions could be used to explore why respondents shared such strong beliefs regarding the importance of the Top Tier ‘core’ reasons for partnership success.

Our approach can be used as an evaluation technique before, during, and after partnerships to measure participants’ belief systems, set partnership goals, establish benchmarks for success, and plan partnership activities. For example, participants can use our approach to measure their shared beliefs at the outset of a partnership and then periodically throughout the partnership to determine if participants’ beliefs change. Participants can use these results to adjust partnership steps and activities as needed. This effort can then be replicated as part of a final partnership evaluation to determine how beliefs and mental models compare across the partnership’s life cycle. This approach is not limited to questions about reasons for success, it can be used to measure shared beliefs regarding other partnership design elements.

By sampling people with academic-nonprofit experience, we measured beliefs related to a larger distributed cognition regarding these partnerships. However, this approach did not capture the connection between shared beliefs regarding partnership success and successful partnership outcomes. In order to measure team cognition and determine if there is a linkage between shared beliefs and partnership success, this research would need to be replicated with individual partnerships over time. With longitudinal data across multiple partnerships, scholars could measure the association between shared partnership beliefs and the likelihood of partnership success. By understanding people’s shared beliefs regarding how partnerships work, we may be able to better use the scarce resources that make research possible and harness the forces that drive innovation and knowledge creation.

## Supporting information

S1 Fig Q10 Respondent Cultural Competence and 2^nd^ Factor Loading Scores (Gender). (TIFF)

S2 Fig Q10 Respondent Cultural Competence and 2^nd^ Factor Loading Scores (Student Status). (TIFF)

S3 Fig Q10 Respondent Cultural Competence and 2^nd^ Factor Loading Scores (Organization Type). (TIFF)

S4 Fig Q10 Respondent Cultural Competence and 2^nd^ Factor Loading Scores (Experience Type). (TIFF)

S5 Fig Q10 Respondent Cultural Competence and 2^nd^ Factor Loading Scores (Above Median Number of Partnerships). (TIFF)

S6 Fig Q10 Respondent Cultural Competence and 2^nd^ Factor Loading Scores (Above Median Number of Years Partnerships). (TIFF)

S7 Fig Q10 Answer Key Divergence (Student vs Non-student). (TIFF)

S8 Fig Q10 Answer Key Divergence (Academic vs Nonprofit). (TIFF)

S9 Fig Q10 Answer Key Divergence (Academic Non-student vs Nonprofit Non-student). (TIFF)

S10 Fig Q10 Answer Key Divergence (Academic vs Nonprofit Experience). (TIFF)

S11 Fig Q10 Answer Key Divergence (Academic vs Both Experience). (TIFF)

S12 Fig Q10 Answer Key Divergence (Nonprofit vs Both Experience). (TIFF)

S13 Fig Q10 Answer Key Divergence (Above vs Below Median Number of Partnerships). (TIFF)

S14 Fig Q10 Answer Key Divergence (Above vs Below Median Number of Partnership Years). (TIFF)

S1 Table A Reasons for academic-nonprofit partnership success from literature review and semi-structured interviews. This table contains our list of reasons for partnership success based on a literature review and semi-structured interviews.
(DOCX)

S1 Table B Post hoc pairwise comparisons Conover test with a Bonferroni correction. Top-Tier and Mid-High Tier reasons in bold. This table provides the results of Conover test and the resulting p-values.
(DOCX)

S1 Table C Marginal ranking table of reasons for academic-nonprofit partnership success. This table shows how often each reason for partnership success received each ranking.
(DOCX)

S1 Table D Kendall’s Tau correlation table of cultural consensus subgroup answer keys. This table shows the intensity of the correlations of rankings across subgroup cultural consensus answer keys.
(DOCX)
