# Supplementary material for: What makes partnerships work? Measuring agreement in participant beliefs regarding ‘core’ reasons for success
Source: PLoS One. 2026 Sep 18;21(9):e0356371. doi: 10.1371/journal.pone.0356371 (PMC13588532; doi:10.1371/journal.pone.0356371)
Supplement: Supplementary file 1 [file pone.0356371.s015.docx]

| **ID** | **Reasons for success** | **References** | **Interview mentions** |
| --- | --- | --- | --- |
| Q10b | Clear partnership and project goals, expectations, deliverables, and outcomes | (Drahota et al., 2016; Eddy, 2010; Grobe, 1993; Lapierre et al., 2018; Wildridge et al., 2004) | 25 (56.8%) |
| Q10l | Participants have a shared partnership vision/purpose | (Drahota et al., 2016; Eddy, 2010; Gomez et al., 2021; Jozefowicz, 2015; Wildridge et al., 2004) | 20 (45.5%) |
| Q10k | Regular and clear communication | (Drahota et al., 2016; Gomez et al., 2021; Grobe, 1993; Wildridge et al., 2004) | 16 (36.4%) |
| Q10q | Sufficient funding to complete partnership activities and tasks | (Ross et al., 2010; Wildridge et al., 2004; Wolff & Maurana, 2001) | 13 (29.5%) |
| Q10e | Shared decision-making opportunities | (Eddy, 2010; Grobe, 1993; Hailes et al., 2022; Wildridge et al., 2004; Wolff & Maurana, 2001) | 11 (25%) |
| Q10h | Shared feelings of influence and power in partnership decisions | (Hailes et al., 2022; Kanj, 2024; Wolff & Maurana, 2001) | 11 (25%) |
| Q10c | Equitable distribution of partnership benefits | (Drahota et al., 2016; Gomez et al., 2021; Grobe, 1993) | 10 (22.7%) |
| Q10a | Realistic timelines to complete partnership tasks | (Grobe, 1993; Lapierre et al., 2018) | 7 (15.9%) |
| Q10o | Clear partner roles and responsibilities | (Gomez et al., 2021; Grobe, 1993; Lapierre et al., 2018; Ross et al., 2010; Wildridge et al., 2004; Wolff & Maurana, 2001) | 7 (15.9%) |
| Q10p | Partnership champion (e.g. an influential person who can advocate for the partnership with decision-makers) | (Eddy, 2010; Grobe, 1993) | 7 (15.9%) |
| Q10d | Administrative buy-in and support from decision-makers | (Eddy, 2010; Grobe, 1993; Lapierre et al., 2018) | 6 (13.6%) |
| Q10j | Clear partnership duration and end date | (Ross et al., 2010) | 2 (4.5%) |
| Q10m | Co-developed partnership language that avoids jargon | (Drahota et al., 2016; Gomez et al., 2021) | 2 (4.5%) |
| Q10f | Having a system to monitor and measure partnership activities | (Grobe, 1993; Wildridge et al., 2004) | 1 (2.3%) |
| Q10g | Equal distribution of tasks and responsibilities among partners | (Ross et al., 2010; Wolff & Maurana, 2001) | 1 (2.3%) |
| Q10i | Equitable investment of resources | (Grant, 2022; Wolff & Maurana, 2001) | 1 (2.3%) |
| Q10n | Partnership risks are shared equitably | (Ross et al., 2010) | 1 (2.3%) |
| Q10r | Establishing a co-created conflict resolution plan | (Drahota et al., 2016; Gomez et al., 2021; Ross et al., 2010) | 1 (2.3%) |

S1 Table A. Reasons for academic-nonprofit partnership success from literature review and semi-structured interviews mentions of academic partnership success reasons

**Supplentary Table A References**

Drahota, A., Meza, R. D., Brikho, B., Naaf, M., Estabillo, J. A., Gomez, E. D., Vejnoska, S. F., Dufek, S., Stahmer, A. C., & Aarons, G. A. (2016). Community-Academic Partnerships: A Systematic Review of the State of the Literature and Recommendations for Future Research. *The Milbank Quarterly*, *94*(1), 163-214. https://doi.org/10.1111/1468-0009.12184

Eddy, P. L. (2010). *Partnerships and Collaborations in Higher Education*. Wiley Periodicals, Inc.

Gomez, E., Drahota, A., & Stahmer, A. C. (2021). Choosing strategies that work from the start: A mixed methods study to understand effective development of community–academic partnerships. *Action Research*, 19(2), 277-300. <https://doi.org/10.1177/1476750318775796>

Grant, C. (2022). Collaborative Tactics for Equitable Community Partnerships Toward Social Justice Impact. *IEEE Transactions on Professional Communication*, 65(1). https://doi.org/10.1109/TPC.2022.3141227

Grobe, T. (1993). *Synthesis of Existing Knowledge and Practice in the Field of Educational Partnerships*. US Department of Education.

Hailes, H. P., Colgan, C., Goodman, L. A., & Thomas, K. A. (2022). Sustaining Long-term Community-Academic Partnerships: Negotiating Power and Presence. *Journal for Social Action in Counseling & Psychology*, 14(2), 81-95. https://doi.org/10.33043/JSACP.14.2.81-95

Jozefowicz, V. L. (2015). *A Study Of Factors That Influence Partnerships Between Universities And Nonprofit Organizations* [PhD, Eastern Kentucky University].

Kanj, M. (2024). "One Day It Will Happen For Us": The Power of Academia in Community-University Service-Learning Partnerships. *Journal of Community Engagement & Scholarship*, 17(1), 1-13. https://doi.org/10.54656/jces.v17i1.555

Lapierre, L. M., Matthews, R. A., Eby, L. T., Truxillo, D. M., Johnson, R. E., & Major, D. A. (2018). Recommended Practices for Academics to Initiate and Manage Research Partnerships With Organizations. *Industrial and Organizational Psychology*, 11(4), 543-581. https://doi.org/10.1017/iop.2018.83

Ross, L. F., Loup, A., Nelson, R. M., Botkin, J. R., Kost, R., Smith, J., George R., & Gehlert, S. (2010). The Challenges of Collaboration for Academic and Community Partners in a Research Partnership: Points to Consider. *Journal of Empirical Research on Human Research Ethics*, 19-31. https://doi.org/10.1525/jer.2010.5.1.19

Wildridge, V., Childs, S., Cawthra, L., & Madge, B. (2004). How to create successful partnerships—a review of the literature. *Health Information and Libraries Journal*, 21, 3-19.

Wolff, M., & Maurana, C. A. (2001). Building Effective Community–Academic Partnerships to Improve Health: A Qualitative Study of Perspectives from Communities. *Academic Medicine*, 76(2), 166-172.
