# Supplementary material for: What makes partnerships work? Measuring agreement in participant beliefs regarding ‘core’ reasons for success
Source: PLoS One. 2026 Sep 18;21(9):e0356371. doi: 10.1371/journal.pone.0356371 (PMC13588532; doi:10.1371/journal.pone.0356371)
Supplement: Supplementary file 2 [file pone.0356371.s016.docx]

S1 Table B. Post hoc pairwise comparisons Conover test with a Bonferroni correction. Top-Tier and Mid-High Tier reasons in bold.

|  | Comparison | Conover Statistic (t) | Adjusted p-value |
| --- | --- | --- | --- |
| 1 | **Q10b** vs. Q10a | 7.34 | < 0.001 |
| 2 | Q10c vs. Q10a | -0.32 | 1 |
| 3 | Q10c vs. **Q10b** | -7.67 | < 0.001 |
| 4 | Q10d vs. Q10a | 2.01 | 1 |
| 5 | Q10d vs. **Q10b** | -5.33 | < 0.001 |
| 6 | Q10d vs. Q10c | 2.33 | 1 |
| 7 | Q10e vs. Q10a | 1.43 | 1 |
| 8 | Q10e vs. **Q10b** | -5.91 | < 0.001 |
| 9 | Q10e vs. Q10c | 1.76 | 1 |
| 10 | Q10e vs. Q10d | -0.58 | 1 |
| 11 | Q10f vs. Q10a | -1.24 | 1 |
| 12 | Q10f vs. **Q10b** | -8.59 | < 0.001 |
| 13 | Q10f vs. Q10c | -0.92 | 1 |
| 14 | Q10f vs. Q10d | -3.25 | 0.178 |
| 15 | Q10f vs. Q10e | -2.68 | 1 |
| 16 | Q10g vs. Q10a | -2.16 | 1 |
| 17 | Q10g vs. **Q10b** | -9.51 | < 0.001 |
| 18 | Q10g vs. Q10c | -1.84 | 1 |
| 19 | Q10g vs. Q10d | -4.17 | 0.005 |
| 20 | Q10g vs. Q10e | -3.6 | 0.051 |
| 21 | Q10g vs. Q10f | -0.92 | 1 |
| 22 | Q10h vs. Q10a | 0.66 | 1 |
| 23 | Q10h vs. **Q10b** | -6.68 | < 0.001 |
| 24 | Q10h vs. Q10c | 0.99 | 1 |
| 25 | Q10h vs. Q10d | -1.35 | 1 |
| 26 | Q10h vs. Q10e | -0.77 | 1 |
| 27 | Q10h vs. Q10f | 1.91 | 1 |
| 28 | Q10h vs. Q10g | 2.83 | 0.726 |
| 29 | Q10i vs. Q10a | -1.98 | 1 |
| 30 | Q10i vs. **Q10b** | -9.32 | < 0.001 |
| 31 | Q10i vs. Q10c | -1.65 | 1 |
| 32 | Q10i vs. Q10d | -3.99 | 0.011 |
| 33 | Q10i vs. Q10e | -3.41 | 0.103 |
| 34 | Q10i vs. Q10f | -0.73 | 1 |
| 35 | Q10i vs. Q10g | 0.19 | 1 |
| 36 | Q10i vs. Q10h | -2.64 | 1 |
| 37 | Q10j vs. Q10a | -3.24 | 0.189 |
| 38 | Q10j vs. **Q10b** | -10.58 | < 0.001 |
| 39 | Q10j vs. Q10c | -2.91 | 0.555 |
| 40 | Q10j vs. Q10d | -5.25 | < 0.001 |
| 41 | Q10j vs. Q10e | -4.67 | < 0.001 |
| 42 | Q10j vs. Q10f | -1.99 | 1 |
| 43 | Q10j vs. Q10g | -1.07 | 1 |
| 44 | Q10j vs. Q10h | -3.9 | 0.015 |
| 45 | Q10j vs. Q10i | -1.26 | 1 |
| 46 | **Q10k** vs. Q10a | 6.37 | < 0.001 |
| 47 | **Q10k** vs. **Q10b** | -0.97 | 1 |
| 48 | **Q10k** vs. Q10c | 6.7 | < 0.001 |
| 49 | **Q10k** vs. Q10d | 4.36 | 0.002 |
| 50 | **Q10k** vs. Q10e | 4.94 | < 0.001 |
| 51 | **Q10k** vs. Q10f | 7.62 | < 0.001 |
| 52 | **Q10k** vs. Q10g | 8.54 | < 0.001 |
| 53 | **Q10k** vs. Q10h | 5.71 | < 0.001 |
| 54 | **Q10k** vs. Q10i | 8.35 | < 0.001 |
| 55 | **Q10k** vs. Q10j | 9.61 | < 0.001 |
| 56 | **Q10l** vs. Q10a | 6.9 | < 0.001 |
| 57 | **Q10l** vs. **Q10b** | -0.44 | 1 |
| 58 | **Q10l** vs. Q10c | 7.23 | < 0.001 |
| 59 | **Q10l** vs. Q10d | 4.89 | < 0.001 |
| 60 | **Q10l** vs. Q10e | 5.47 | < 0.001 |
| 61 | **Q10l** vs. Q10f | 8.15 | < 0.001 |
| 62 | **Q10l** vs. Q10g | 9.07 | < 0.001 |
| 63 | **Q10l** vs. Q10h | 6.24 | < 0.001 |
| 64 | **Q10l** vs. Q10i | 8.88 | < 0.001 |
| 65 | **Q10l** vs. Q10j | 10.14 | < 0.001 |
| 66 | **Q10l** vs. **Q10k** | 0.53 | 1 |
| 67 | Q10m vs. Q10a | -3.41 | 0.103 |
| 68 | Q10m vs. **Q10b** | -10.75 | < 0.001 |
| 69 | Q10m vs. Q10c | -3.08 | 0.319 |
| 70 | Q10m vs. Q10d | -5.42 | < 0.001 |
| 71 | Q10m vs. Q10e | -4.84 | < 0.001 |
| 72 | Q10m vs. Q10f | -2.16 | 1 |
| 73 | Q10m vs. Q10g | -1.24 | 1 |
| 74 | Q10m vs. Q10h | -4.07 | 0.008 |
| 75 | Q10m vs. Q10i | -1.43 | 1 |
| 76 | Q10m vs. Q10j | -0.17 | 1 |
| 77 | Q10m vs. **Q10k** | -9.78 | < 0.001 |
| 78 | Q10m vs. **Q10l** | -10.31 | < 0.001 |
| 79 | Q10n vs. Q10a | -3.65 | 0.042 |
| 80 | Q10n vs. **Q10b** | -10.99 | < 0.001 |
| 81 | Q10n vs. Q10c | -3.32 | 0.14 |
| 82 | Q10n vs. Q10d | -5.66 | < 0.001 |
| 83 | Q10n vs. Q10e | -5.08 | < 0.001 |
| 84 | Q10n vs. Q10f | -2.4 | 1 |
| 85 | Q10n vs. Q10g | -1.48 | 1 |
| 86 | Q10n vs. Q10h | -4.31 | 0.003 |
| 87 | Q10n vs. Q10i | -1.67 | 1 |
| 88 | Q10n vs. Q10j | -0.41 | 1 |
| 89 | Q10n vs. **Q10k** | -10.02 | < 0.001 |
| 90 | Q10n vs. **Q10l** | -10.55 | < 0.001 |
| 91 | Q10n vs. Q10m | -0.24 | 1 |
| 92 | **Q10o** vs. Q10a | 3.99 | 0.011 |
| 93 | **Q10o** vs. **Q10b** | -3.36 | 0.124 |
| 94 | **Q10o** vs. Q10c | 4.31 | 0.003 |
| 95 | **Q10o** vs. Q10d | 1.98 | 1 |
| 96 | **Q10o** vs. Q10e | 2.56 | 1 |
| 97 | **Q10o** vs. Q10f | 5.23 | < 0.001 |
| 98 | **Q10o** vs. Q10g | 6.15 | < 0.001 |
| 99 | **Q10o** vs. Q10h | 3.32 | 0.14 |
| 100 | **Q10o** vs. Q10i | 5.96 | < 0.001 |
| 101 | **Q10o** vs. Q10j | 7.23 | < 0.001 |
| 102 | **Q10o** vs. **Q10k** | -2.39 | 1 |
| 103 | **Q10o** vs. **Q10l** | -2.91 | 0.555 |
| 104 | **Q10o** vs. Q10m | 7.4 | < 0.001 |
| 105 | **Q10o** vs. Q10n | 7.63 | < 0.001 |
| 106 | Q10p vs. Q10a | 0.07 | 1 |
| 107 | Q10p vs. **Q10b** | -7.28 | < 0.001 |
| 108 | Q10p vs. Q10c | 0.39 | 1 |
| 109 | Q10p vs. Q10d | -1.94 | 1 |
| 110 | Q10p vs. Q10e | -1.36 | 1 |
| 111 | Q10p vs. Q10f | 1.31 | 1 |
| 112 | Q10p vs. Q10g | 2.23 | 1 |
| 113 | Q10p vs. Q10h | -0.6 | 1 |
| 114 | Q10p vs. Q10i | 2.04 | 1 |
| 115 | Q10p vs. Q10j | 3.31 | 0.149 |
| 116 | Q10p vs. **Q10k** | -6.3 | < 0.001 |
| 117 | Q10p vs. **Q10l** | -6.83 | < 0.001 |
| 118 | Q10p vs. Q10m | 3.48 | 0.08 |
| 119 | Q10p vs. Q10n | 3.71 | 0.032 |
| 120 | Q10p vs. **Q10o** | -3.92 | 0.014 |
| 121 | **Q10q** vs. Q10a | 3.41 | 0.103 |
| 122 | **Q10q** vs. **Q10b** | -3.94 | 0.013 |
| 123 | **Q10q** vs. Q10c | 3.73 | 0.03 |
| 124 | **Q10q** vs. Q10d | 1.4 | 1 |
| 125 | **Q10q** vs. Q10e | 1.98 | 1 |
| 126 | **Q10q** vs. Q10f | 4.65 | < 0.001 |
| 127 | **Q10q** vs. Q10g | 5.57 | < 0.001 |
| 128 | **Q10q** vs. Q10h | 2.74 | 0.943 |
| 129 | **Q10q** vs. Q10i | 5.38 | < 0.001 |
| 130 | **Q10q** vs. Q10j | 6.65 | < 0.001 |
| 131 | **Q10q** vs. **Q10k** | -2.96 | 0.471 |
| 132 | **Q10q** vs. **Q10l** | -3.49 | 0.075 |
| 133 | **Q10q** vs. Q10m | 6.82 | < 0.001 |
| 134 | **Q10q** vs. Q10n | 7.05 | < 0.001 |
| 135 | **Q10q** vs. **Q10o** | -0.58 | 1 |
| 136 | **Q10q** vs. Q10p | 3.34 | 0.132 |
| 137 | Q10r vs. Q10a | -3.92 | 0.014 |
| 138 | Q10r vs. **Q10b** | -11.26 | < 0.001 |
| 139 | Q10r vs. Q10c | -3.6 | 0.051 |
| 140 | Q10r vs. Q10d | -5.93 | < 0.001 |
| 141 | Q10r vs. Q10e | -5.35 | < 0.001 |
| 142 | Q10r vs. Q10f | -2.68 | 1 |
| 143 | Q10r vs. Q10g | -1.76 | 1 |
| 144 | Q10r vs. Q10h | -4.58 | < 0.001 |
| 145 | Q10r vs. Q10i | -1.94 | 1 |
| 146 | Q10r vs. Q10j | -0.68 | 1 |
| 147 | Q10r vs. **Q10k** | -10.29 | < 0.001 |
| 148 | Q10r vs. **Q10l** | -10.82 | < 0.001 |
| 149 | Q10r vs. Q10m | -0.51 | 1 |
| 150 | Q10r vs. Q10n | -0.27 | 1 |
| 151 | Q10r vs. **Q10o** | -7.91 | < 0.001 |
| 152 | Q10r vs. Q10p | -3.99 | 0.011 |
| 153 | Q10r vs. **Q10q** | -7.33 | < 0.001 |
