# Supplementary material for: What makes partnerships work? Measuring agreement in participant beliefs regarding ‘core’ reasons for success
Source: PLoS One. 2026 Sep 18;21(9):e0356371. doi: 10.1371/journal.pone.0356371 (PMC13588532; doi:10.1371/journal.pone.0356371)
Supplement: Supplementary file 3 [file pone.0356371.s017.docx]

S1 Table C. Marginal ranking table of reasons for academic-nonprofit partnership success.

|  | Q10a | Q10b | Q10c | Q10d | Q10e | Q10f | Q10g | Q10h | Q10i | Q10j | Q10k | Q10l | Q10m | Q10n | Q10o | Q10p | Q10q | Q10r |
| --- | --- | --- | --- | --- | --- | --- | --- | --- | --- | --- | --- | --- | --- | --- | --- | --- | --- | --- |
| Rank1 | 0 | 16 | 1 | 3 | 3 | 3 | 1 | 2 | 2 | 0 | 7 | 29 | 0 | 0 | 2 | 5 | 6 | 0 |
| Rank2 | 1 | 18 | 4 | 5 | 3 | 2 | 1 | 5 | 1 | 1 | 10 | 7 | 0 | 1 | 3 | 7 | 9 | 2 |
| Rank3 | 4 | 12 | 2 | 9 | 4 | 1 | 1 | 4 | 3 | 1 | 9 | 7 | 2 | 0 | 8 | 2 | 10 | 1 |
| Rank4 | 4 | 7 | 3 | 2 | 4 | 2 | 5 | 3 | 4 | 1 | 9 | 6 | 2 | 1 | 12 | 5 | 6 | 4 |
| Rank5 | 4 | 4 | 5 | 11 | 5 | 3 | 2 | 5 | 1 | 3 | 6 | 3 | 6 | 0 | 11 | 4 | 6 | 1 |
| Rank6 | 3 | 4 | 5 | 6 | 7 | 5 | 5 | 5 | 1 | 3 | 11 | 1 | 3 | 5 | 9 | 3 | 3 | 1 |
| Rank7 | 5 | 5 | 3 | 5 | 5 | 3 | 2 | 6 | 4 | 2 | 12 | 4 | 2 | 2 | 8 | 4 | 4 | 4 |
| Rank8 | 7 | 2 | 4 | 7 | 5 | 7 | 5 | 3 | 5 | 5 | 3 | 4 | 3 | 3 | 5 | 2 | 7 | 3 |
| Rank9 | 9 | 0 | 9 | 1 | 8 | 4 | 2 | 4 | 6 | 5 | 5 | 5 | 5 | 5 | 4 | 3 | 3 | 2 |
| Rank10 | 6 | 2 | 6 | 2 | 7 | 6 | 4 | 7 | 5 | 4 | 4 | 2 | 4 | 5 | 3 | 6 | 4 | 3 |
| Rank11 | 9 | 1 | 5 | 3 | 8 | 6 | 4 | 6 | 3 | 7 | 2 | 2 | 4 | 8 | 2 | 3 | 2 | 5 |
| Rank12 | 5 | 3 | 8 | 3 | 6 | 6 | 6 | 6 | 6 | 10 | 1 | 3 | 2 | 4 | 1 | 6 | 1 | 3 |
| Rank13 | 5 | 1 | 4 | 3 | 0 | 5 | 11 | 9 | 7 | 2 | 1 | 3 | 7 | 5 | 3 | 4 | 4 | 6 |
| Rank14 | 4 | 2 | 1 | 4 | 4 | 4 | 7 | 4 | 6 | 8 | 0 | 1 | 6 | 13 | 2 | 6 | 2 | 6 |
| Rank15 | 8 | 0 | 7 | 7 | 4 | 3 | 7 | 1 | 6 | 7 | 0 | 1 | 8 | 5 | 2 | 1 | 5 | 8 |
| Rank16 | 3 | 2 | 4 | 2 | 3 | 10 | 6 | 2 | 10 | 3 | 0 | 0 | 3 | 8 | 2 | 5 | 4 | 13 |
| Rank17 | 2 | 0 | 5 | 4 | 1 | 7 | 7 | 4 | 6 | 3 | 0 | 1 | 10 | 11 | 2 | 6 | 2 | 9 |
| Rank18 | 1 | 1 | 4 | 3 | 3 | 3 | 4 | 4 | 4 | 15 | 0 | 1 | 13 | 4 | 1 | 8 | 2 | 9 |
