# Supplementary material for: What makes partnerships work? Measuring agreement in participant beliefs regarding ‘core’ reasons for success
Source: PLoS One. 2026 Sep 18;21(9):e0356371. doi: 10.1371/journal.pone.0356371 (PMC13588532; doi:10.1371/journal.pone.0356371)
Supplement: Supplementary file 4 [file pone.0356371.s018.docx]

| Keys | A* | NS* | S | AO | NO | ANS | NNS* | AEO | NEO | BE* | BENSTO* | BENSAO | AMN* | BMN | AMNY* | BMNY |
| --- | --- | --- | --- | --- | --- | --- | --- | --- | --- | --- | --- | --- | --- | --- | --- | --- |
| A* |  | 0.96**** | 0.59*** | 0.92**** | 0.88**** | 0.93**** | 0.88**** | 0.78**** | 0.86**** | 0.86**** | 0.87**** | 0.86**** | 0.93**** | 0.9**** | 0.91**** | 0.8**** |
| NS* | 0.96**** |  | 0.56*** | 0.91**** | 0.9**** | 0.95**** | 0.9**** | 0.76**** | 0.87**** | 0.87**** | 0.88**** | 0.87**** | 0.97**** | 0.86**** | 0.95**** | 0.76**** |
| S | 0.59*** | 0.56*** |  | 0.54** | 0.56*** | 0.56*** | 0.56*** | 0.69**** | 0.58*** | 0.5** | 0.49** | 0.5** | 0.53** | 0.62*** | 0.53** | 0.61*** |
| AO | 0.92**** | 0.91**** | 0.54** |  | 0.8**** | 0.93**** | 0.8**** | 0.8**** | 0.78**** | 0.86**** | 0.84**** | 0.86**** | 0.88**** | 0.87**** | 0.88**** | 0.8**** |
| NO | 0.88**** | 0.9**** | 0.56*** | 0.8**** |  | 0.84**** | 1**** | 0.74**** | 0.87**** | 0.82**** | 0.83**** | 0.79**** | 0.92**** | 0.91**** | 0.84**** | 0.79**** |
| ANS | 0.93**** | 0.95**** | 0.56*** | 0.93**** | 0.84**** |  | 0.84**** | 0.79**** | 0.82**** | 0.84**** | 0.86**** | 0.84**** | 0.92**** | 0.83**** | 0.92**** | 0.76**** |
| NNS* | 0.88**** | 0.9**** | 0.56*** | 0.8**** | 1**** | 0.84**** |  | 0.74**** | 0.87**** | 0.82**** | 0.83**** | 0.79**** | 0.92**** | 0.91**** | 0.84**** | 0.79**** |
| AEO | 0.78**** | 0.76**** | 0.69**** | 0.8**** | 0.74**** | 0.79**** | 0.74**** |  | 0.69**** | 0.69**** | 0.7**** | 0.66**** | 0.74**** | 0.78**** | 0.74**** | 0.66**** |
| NEO | 0.86**** | 0.87**** | 0.58*** | 0.78**** | 0.87**** | 0.82**** | 0.87**** | 0.69**** |  | 0.74**** | 0.75**** | 0.74**** | 0.84**** | 0.86**** | 0.87**** | 0.69**** |
| BE* | 0.86**** | 0.87**** | 0.5** | 0.86**** | 0.82**** | 0.84**** | 0.82**** | 0.69**** | 0.74**** |  | 0.96**** | 0.92**** | 0.9**** | 0.78**** | 0.82**** | 0.82**** |
| BENSTO* | 0.87**** | 0.88**** | 0.49** | 0.84**** | 0.83**** | 0.86**** | 0.83**** | 0.7**** | 0.75**** | 0.96**** |  | 0.91**** | 0.91**** | 0.76**** | 0.83**** | 0.8**** |
| BENSAO | 0.86**** | 0.87**** | 0.5** | 0.86**** | 0.79**** | 0.84**** | 0.79**** | 0.66**** | 0.74**** | 0.92**** | 0.91**** |  | 0.87**** | 0.78**** | 0.82**** | 0.76**** |
| AMN* | 0.93**** | 0.97**** | 0.53** | 0.88**** | 0.92**** | 0.92**** | 0.92**** | 0.74**** | 0.84**** | 0.9**** | 0.91**** | 0.87**** |  | 0.83**** | 0.92**** | 0.79**** |
| BMN | 0.9**** | 0.86**** | 0.62*** | 0.87**** | 0.91**** | 0.83**** | 0.91**** | 0.78**** | 0.86**** | 0.78**** | 0.76**** | 0.78**** | 0.83**** |  | 0.8**** | 0.78**** |
| AMNY* | 0.91**** | 0.95**** | 0.53** | 0.88**** | 0.84**** | 0.92**** | 0.84**** | 0.74**** | 0.87**** | 0.82**** | 0.83**** | 0.82**** | 0.92**** | 0.8**** |  | 0.71**** |
| BMNY | 0.8**** | 0.76**** | 0.61*** | 0.8**** | 0.79**** | 0.76**** | 0.79**** | 0.66**** | 0.69**** | 0.82**** | 0.8**** | 0.76**** | 0.79**** | 0.78**** | 0.71**** |  |

S1 Table D. Kendall’s Tau correlation table of cultural consensus subgroup answer keys.

A* = All	NEO = Nonprofit Experience Only	* = p < 0.05

NS* = Non-student	BEO* = Both Experience	** = p < 0.01

S = Student	BENSTO* = Both Experience Non-student Only	*** = p < 0.001

AO = Academic Organization	BENSAO = Both Experience Non-student Only	**** = p < 0.0001

NO = Nonprofit Organization	AMN* = Above Median Number of Partnerships

ANS = Academic Non-student	BMN = Below Median Number of Partnerships

NNS* = Nonprofit Non-student	AMNY* = Above Median Number of Years Partnerships

AEO = Academic Experience Only	BMNY = Below Median Number of Years Partnership
